# Antimicrobial Poly (Lactic Acid)/Copper Nanocomposites for Food Packaging Materials

**DOI:** 10.3390/ma16041415

**Published:** 2023-02-08

**Authors:** Violeta Popescu, Doina Prodan, Stanca Cuc, Codruţa Saroşi, Gabriel Furtos, Andrei Moldovan, Rahela Carpa, Dorin Bomboş

**Affiliations:** 1 Faculty of Materials Engineering and the Environment, Technical University of Cluj-Napoca, Bd. Muncii 103-105, 400641 Cluj-Napoca, Romania; 2 Raluca Ripan Institute of Research in Chemistry, Babes Bolyai University, 30 Fantanele Street, 400294 Cluj-Napoca, Romania; 3 Department of Molecular Biology and Biotechnology, Faculty of Biology and Geology, Babes Bolyai University, 1 M. Kogalniceanu Street, 400084 Cluj-Napoca, Romania; 4 S.C. Medacril S.R.L, 8 Carpați Street, Mediaş, 551022 Sibiu, Romania; 5 Petroleum-Gas University of Ploieşti, 39 Bucuresti Blvd., 100680 Ploieşti, Romania

**Keywords:** food packaging, antimicrobial properties, mechanical properties, copper

## Abstract

Composites based on polylactic acid (PLA) and copper for food packaging applications were obtained. Copper clusters were synthesized in polyethylene glycols 400 and 600, respectively, using ascorbic acid as a reducing agent, by reactive milling. Copper clusters were characterized by Scanning Electron Microscopy (SEM), Fourier Transform Infrared (FT-IR), and Ultraviolet-Visible (UV-VIS) spectroscopy. Copper/PLA composites containing Proviplast as plasticizer were characterized by FT-IR spectroscopy, mechanical tests, Differential Scanning Calorimetry (DSC), Thermogravimetric Analysis (TGA), absorption of the saline solution, contact angle, and antibacterial properties. It was observed that the concentration of Copper/PEG influenced the investigated properties. The mechanical properties of the samples decreased with the increasing of Copper/PEG concentration. We recorded the phase transformation temperatures and identified the exothermic or endothermic processes. The lowest absorption values were recorded in the case of the sample containing 1% Cu. The contact angle decreases with the increase in the concentration of the PEG 600-Cu mixture in the recipes. The increase in the content of Cu clusters favors the decrease in the temperature, taking place 15% wt mass losses. The obtained composites showed antibacterial properties for all tested strains. These materials could be used as alternative materials for obtaining biodegradable food packaging.

## 1. Introduction

The major impact of packaging materials on the environment imposes the necessity of the identification of alternative solutions to traditional packaging materials based on petrochemical products. The lifetime of the food packaging materials before reaching wastelands is very short. The legislation in the European Union imposes selective collection and recycling of polymers. Contamination of the materials with food waste makes these materials unsuitable for recycling. This is the reason why, in the last few years, new materials with a smaller impact on the environment, being biodegradable and bioassimilable, tend to replace conventional materials in the food industry [1].

Bioplastics used for food packaging are more expensive than traditional materials based on polyolefins, such as Polyethylene (PE), Polypropylene (PP), Polystyrene (PS), or polyethylene terephthalate (PET), resulting in the necessity of the conception, and testing of new materials which fulfill all the criteria imposed for materials in contact with food products [1,2].

Compared to silver, the biocide activity of copper is smaller (about 10 mg Cu^2+^/kg in water is necessary for killing 10^6^ cells of *Saccharomyces cerevisiae*). Copper is less expensive and can be obtained using green methods [3,4,5,6]. Copper nanoparticles can be used in food packaging materials as functional additives with antibacterial properties [7]. Copper can be found in food as ions or salts at concentrations smaller than 2 mg Cu^2+^_/_kg (meat, fish, pecan nuts, green vegetables) and up to 39 mg Cu^2+^/kg in cocoa and liver [8], playing an important role in the formation of metalloproteins and enzymes [9].

Copper particles can be obtained both by chemical and physical methods. A series of methods of Cu nano-powders obtaining involves reduction reactions of copper (II) salts with diverse reducing agents such as ascorbic acid [10,11], hydrazine [12], hypophosphite [13], sodium borohydride [14,15,16], hydrogen [17], and sodium phosphinate monohydrate (NaH_2_·PO_2_·H_2_O) in diethylene, which has the role of both reaction environment and reducing agent [18], hydrazine borane (BH_4_N_2_) [19], or ascorbic acid and sodium borohydride [10]. Copper compounds used as precursors for metallic copper or other copper derivatives are copper chloride [20,21], copper nitrate (Cu(NO_3_)_2_ × 3H_2_O) [22,23], copper sulphate (CuSO_4_ × 5H_2_O) [20,21,24,25], or copper acetate (Cu(CH_3_COO)_2_ × H_2_O) [20,21,22]. Copper is used in many applications due to its strong antimicrobial properties and due to its economic advantages being less expensive than gold and silver [26].

In the case of copper nanoparticles synthesis in ethylene-glycol, the reducing capacity of this is not sufficient for reducing copper ions because they are easily oxidized to CuO or Cu_2_O by oxygen from the atmosphere [27]. For this reason, Park [18] introduced NaH_2_PO_2_·H_2_O as a supplementary reducing agent. Another method for Cu nano-particles synthesis [10] involved the use of copper sulphate solution with ascorbic acid and sodium borohydride. The reaction is complete at a molar ratio of CuSO_4_: Acid Ascorbic: NaBH_4_ 1:2:10. Due to the efficiency, and the fact that it is an easy method, chemical reduction is one of the preferred methods, and the size of the nanoparticles can be influenced by variables such as pH and temperature, time, or the concentration of metal ions [26].

Food packaging materials based on polylactic acid (PLA) are the most promising bioplastics because they are biodegradable and bio-based and can be used both for flexible and rigid food packaging. Additionally, the PLA industrial price is becoming closer to the price of polyethylene, polypropylene, or polyethylene terephthalate [28,29]. Due to its inactivity, polyethylene glycol (PEG) can act as a carrier, being able to protect Cu clusters by coating them [30]. An important problem in the synthesis of Cu-based particles is the tendency of the particles to agglomerate, which can be prevented by including some polymers used as coating agents. Thus, covalent bonds are formed between the surface of the nanoparticles and the polymer chains that strengthen the stability of the nanoparticles [31].

The literature data demonstrate an improvement of the PLA properties by incorporating some inorganic nanofillers (silver, zinc oxide, titanium dioxide, copper, etc.). Due to the large specific surface area, and due to the ratio between specific surface and volume, the nanoparticles offer an extended interfacial area, leading to a synergistic effect between polymer and nanoparticles and ensuring an improvement of the mechanical and antimicrobial properties of the nanomaterial [32]. In their study, Mulla et al. believed that obtaining PLA-based materials with inorganic nanoparticles can be achieved by three different methods. The first method, namely in situ polymerization, is based on the use of an initiator, radiation, or heat. In the latest reports in the literature, however, the use of the initiator was abandoned, using L-lactide with silanized nanosilica and montmorillonite for the synthesis of PLA-based materials. The moisture within the polymerization apparatus and lactic acid from L-lactide can lead to the initiation of lactide polymerization with the help of neighboring OH groups [32,33]. The second method is solvent or solution casting. The choice of the solvent in which the nanoparticles and PLA are to be dispersed is essential. Dispersion is carried out separately. The combination of the two mixtures is carried out with the help of ultrasound over a certain period of time. PLA is usually dissolved in organic solvents (benzene, tetrahydrofuran, chlorinated solvents, etc.) but inorganic nanoparticles, due to their hydrophobic character, are very difficult to disperse in polar solvents. To improve the dispersion of inorganic nanoparticles in solvents, several treatments or oxidation should be applied to them that can induce a hydrophilic character [34]. The third method is a melting mixing method, a physical method that is both ecological and cost-effective. It is made with the help of an extruder and, thanks to the heat provided by it, PLA and nanoparticles are mixed directly into the melt. Although it is a safe method, sometimes the PLA matrix or the nanoparticles can be damaged. Additionally, the dispersion of inorganic nanoparticles in the PLA matrix is not always homogeneous [32].

The novelty of our study is in obtaining an alternative material based on PLA and Cu, with a potential antibacterial effect, and in investigation of the influence of the different ratio of PLA and of copper clusters, respectively, on the physical–chemical and mechanical properties of the PLA composites, intended for obtaining the biodegradable food packaging. As plasticizing additives, PEG-400, PEG 600, and Proviplast 2624 were used in order to select the most suitable mixture in which Cu clusters are uniformly dispersed.

## 2. Materials and Methods

### 2.1. Materials

CuSO_4_ × 5H_2_O, ascorbic acid, polyethylene glycol (PEG 400), glycerol (Sigma-Aldrich, Taufkirchen, Germany), and PEG 600 (Merck KGaA, Darmstadt, Germany) were used for the obtaining of copper particles. Polylactic acid (PLA) (NatureWorks LLC under the Ingeo^®^ brand) and tributyl 2-acetylcitrate (Proviplast 2624) (Proviron) were used for obtaining the samples.

#### 2.1.1. Obtaining of the Cu-PEG Clusters

For the obtaining of Cu clusters, 10 g of CuSO_4_ × 5H_2_O was milled. Two grams of copper sulphate powder were mixed with 15 g PEG 400 and milled for one hour. After homogenization, 3 g of ascorbic acid was added to the mixture to obtain copper particles by reactive milling. The obtained gel was kept in the refrigerator to decrease the reaction rate of the formation of copper for 24 h, then it was kept at room temperature. By replacing PEG 400 with PEG 600 and keeping all the other conditions unchanged, the viscosity of the obtained gel increased.

#### 2.1.2. Obtaining of the PLA-Based Composites

In order to obtain the PLACu samples, PLA was mixed in a Brabender Plastograph (Brabender^®^ GmbH & Co. KG, Duisburg, Germany) at 180 °C temperature until melting, after which it was mixed with the plasticizer and Cu clusters dispersed in PEG for 15 min. under a nitrogen atmosphere. With the help of the hydraulic press, a plate with a dimension of 20 × 20 × 2 mm was obtained at 180 °C temperature and 15 atm pressure in 5 min. The composition of the samples and the obtaining conditions are presented in Table 1.

The use of polylactic acid in the preparation of the composite, in the absence of the plasticizer, would not allow an efficient homogenization of the components, the role of the plasticizer being, in the first instance, to reduce the viscosity of PLA for a good homogenization. For this reason, all the articles dealing with the synthesis of PLA-based composites use a plasticizer at a concentration close to that in this paper [1,2].

### 2.2. Characterization of the CuSO_4_-PEG Clusters

#### 2.2.1. Scanning Electron Microscopy (SEM)

Scanning Electron Micrographs for the CuSO_4_-PEG mixture were recorded by Inspect S- SEM microscope (FEI Company, Hillsboro, OR, USA).

#### 2.2.2. Fourier Transform Infrared Spectroscopy (FT-IR)

Fourier transform infrared (FT-IR) spectra of precursors were recorded on Spectrum BX (Perkin Elmer, Waltham, MA, USA) FTIR spectrometer, equipped with ATR accessory (PIKE MIRacle^TM^), with a diamond crystal plate in attenuated total reflection (ATR) mode. To determine the IR spectra of Cu-PEG mixture, the organic phase was removed as much as possible from the surface of the samples, and the Cu particles were placed on the ATR window without further processing.

#### 2.2.3. Ultraviolet-Visible Spectroscopy (UV-VIS)

To determine UV-VIS spectra, the mixture of copper clusters and PEG (400 and 600 respectively) was dispersed in glycerol to obtain a high-density mixture for the limitation of the sedimentation of copper powders. UV-VIS spectra were recorded for the powder suspended in glycerol in 10 mm glass spectroscopic cuvettes in transmission mode with a UV-VIS spectrometer (Lambda 35, Perkin Elmer, Waltham, MA, USA).

### 2.3. Characterization of the PLA Cu Samples

#### 2.3.1. Fourier Transform Infrared (FT-IR)

Fourier transform infrared (FT-IR) spectra of cured samples were recorded on Spectrum BX (Perkin Elmer, Waltham, MA, USA) FTIR spectrometer, equipped with ATR accessory (PIKE MIRacle^TM^).

#### 2.3.2. Tensile Strength Testing

The rectangular specimens specific to this test were subjected to tensile tests using the Lloyd LR5k Plus universal mechanical testing machine (Lloyd Instrumente, Ameteklns, West Sussex, England), with a maximum allowed capacity of 5KN, at a loading force of 0.5 N and a speed of 1 mm/minute at ambient temperature (25 °C), according to the ASTM D638-14, using Nexygen software (version 4.0). All the data are the average of at least seven measurements. The statistical differences between the groups of investigated samples were statistically analyzed using the one-way ANOVA test.

#### 2.3.3. Flexural Strength Testing

The flexural strength was achieved by the 3-point technique, according to ASTM D 790; the data were processed using the Nexygen software (version 4.0). All the data are the average of at least seven measurements. The statistical differences between the groups of investigated samples were statistically analyzed using the one-way ANOVA test.

#### 2.3.4. Differential Scanning Calorimetry (DSC)

The analysis was carried out with the help of a 630e, 700 °C Mettler-Toledo calorimeter (Switzerland). Measurement conditions: aluminum crucible-40 μL; heating speed: 10 °C/min; temperature range 25–200 °C; final landing 0.5 min; atmosphere: nitrogen; flow rate: 80 mL/min.

#### 2.3.5. Thermogravimetric Analysis

The samples were characterized by thermogravimetric analysis using a NETZSCH STA 449C Jupiter simultaneous TGA-DSC (Germany, Selb) with a heating rate of 10 °C/min to 800 °C in a nitrogen atmosphere (99.999% purity, 50 mL/min).

#### 2.3.6. Absorption of the Saline Solution (%)

Absorption is expressed as a percentage by weight increase of a sample according to the ASTM D57-Standard Test Method for Absorption of Plastics.

Working procedure:

The rectangular samples with dimensions of 20 mm length, 10 mm width, and 3 mm thickness were placed in a desiccator at 23 °C until a constant mass value, with a precision of 0.001 g (initial M).

The samples were placed individually in vials with 15 mL of 10% saline solution at a constant temperature of 23 °C. At certain periods of time (24 h, 4, 7, 14 days), the samples were removed from the immersion medium and lightly dried with absorbent paper and weighed (final M).

The absorption percentage is calculated with Equation (1):

(1) $$Wsp=\frac{final\;M-initial\;M}{initial\;M}\times 100$$

For each group of investigated samples, four weight percentage increase measurements were recorded. After recording the values, the average and standard deviation were calculated, statistically analyzing with the help of the one-way ANOVA test and the Tukey test, the differences within each group, depending on the immersion time and the differences between the investigated groups, depending on the amount of water absorbed (*p* value below 0.05 being considered significant statistically).

#### 2.3.7. Contact Angle

The water contact angle was determined using a Drop Shape Analyzer, DSA25 (Hamburg, Germany), at room temperature. A drop of 20 μL of distilled water was placed on the surface of the samples and, after the stabilization period (30 s), the image was recorded, and the contact angle was measured with a dedicated software.

#### 2.3.8. Antimicrobial Activity

The microorganisms tested in this study were: *Enterococcus faecalis* ATCC 29212, *Escherichia coli* ATCC 25922, *Staphylococcus aureus* ATCC 25923, and *Pseudomonas aeruginosa* ATCC 27853, from the collection of the Microbiology Laboratory, Faculty of Biology and Geology, Babeş-Bolyai University of Cluj-Napoca.

Each bacterial strain was grown for 24 h on a Nutrient Agar medium [35]. Following that, a dilution of 0.5 McFarland was made from each strain in sterile physiological serum. From these dilutions, each Petri dish was inoculated with the help of a sterile swab soaked in the 0.5 McFarland microbial suspension, spreading over the entire surface of the solid culture medium (Mueller Hinton-Oxoid).

The inoculated Petri dishes were dried for 20 min at 37 °C. Then, with sterile tweezers, the samples were cut in the form of a square of approximately 5 mm and were aseptically taken and applied to the solid culture medium.

Incubation was done for 48 h at 37 °C. The reading was done by measuring the size of the inhibition zone (x): the larger the size of the inhibition zone, the greater the sensitivity of the bacteria to the respective antibacterial substances [36].

## 3. Results and Discussion

### 3.1. The Formation of Copper Particles

Chemical reduction starting from copper salt can be successfully used for obtaining copper and copper derivatives. Among reducing agents, ascorbic acid can be used as a single reagent or in combination with other reducing agents. In order to prevent the agglomeration of copper nanoparticles due to van der Waals attraction forces, the formed copper particles should be covered with polyols or other surface-active agents, assuring the stability of copper powders.

The possible mechanism of the reduction of copper (II) salts with ascorbic acid in aqueous media, in the presence of polyvinyl pyrrolidone and NaOH, was discussed by Liu Ching Ming [37]:


Cu^2+^ → Cu

Cu^2+^ → Cu(OH)_2_ → Cu_2_O → Cu

Cu^2+^ → Cu(OH)_2_ → CuO → Cu_2_O → Cu

Cu^2+^ → Cu(OH)_2_ → Cu

They demonstrated that in the first step, copper ions were converted to Cu(OH)_2_, and then copper hydroxide reacts with ascorbic acid (C_6_H_8_O_6_), in resulting copper and dehydro ascorbic acid (C_6_O_6_H_6_):


Cu(OH)_2_ + C6H_8_O_6_ → Cu_2_O + C_6_H_6_O_6_ + H_2_O

Cu_2_O + C_6_H_8_O_6_ → Cu + C_6_H_6_O_6_ + H_2_

The formation of Cu_2_O in the first step of the reaction has been observed by our team due to the formation of a bright red precipitate that turned to purple and finally to brown–black copper.

Thi My Dung Dang et al. used PEG as a capping agent for obtaining copper nanoparticles [38], but they worked in an aqueous environment. They used both sodium borohydride and ascorbic acid as a reducing and antioxidant agent, respectively. They showed that the size of copper particles depends on the PEG/Cu^2+^ molar ratio—the higher the PEG content, the smaller the sizes of the obtained copper particles. The formation of Cu particles in the presence of PEG also depends on the pH and ascorbic acid concentration [38].

### 3.2. Characterization of the Cu-PEG Clusters

#### 3.2.1. Scanning Electron Microscopy (SEM)

**Figure 1:**
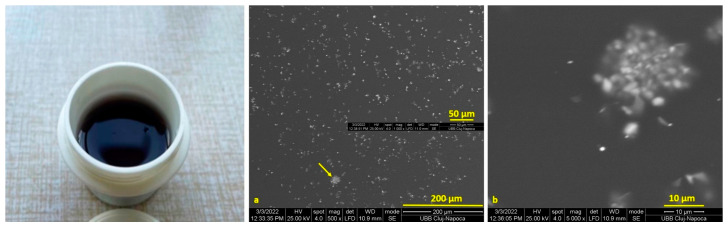
Images of the Cu-PEG 400 mixture (in plastic minicontainer) and (**a**,**b**) SEM images of Cu SO_4_-PEG 400.

**Figure 2:**
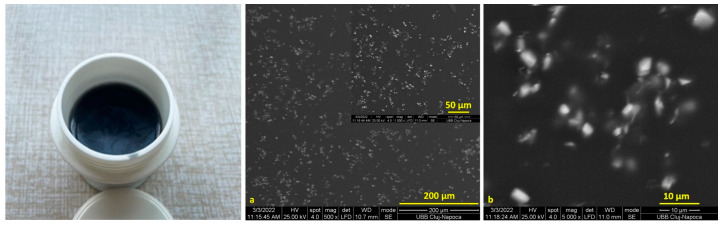
Images of the CuSO4-PEG 600 mixture (in plastic minicontainer) and (**a**,**b**) SEM images of Cu-PEG 600.

SEM images of the Cu-PEG mixture films are presented in Figure 1 and Figure 2. The Cu-PEG 400 and Cu-PEG 600 samples have a gel-like appearance, having a purple color that changes into dark brown after 2–3 days, as can be seen in the plastic minicontainers from Figure 1 and Figure 2. SEM images showed that the CuSO_4_ particles are well-dispersed into PEG400, having some clusters with Cu particles (Figure 1a,b) with sizes up to 10 µm. The Cu-PEG600 sample seems to have a better dispersion of Cu, even if some of the particles tend to agglomerate (Figure 2).

The sample Cu-PEG 600 was selected for obtaining PLA composites because it seems to have a better dispersion and a higher stability.

#### 3.2.2. Fourier Transform Infrared (FT-IR)

**Figure 3:**
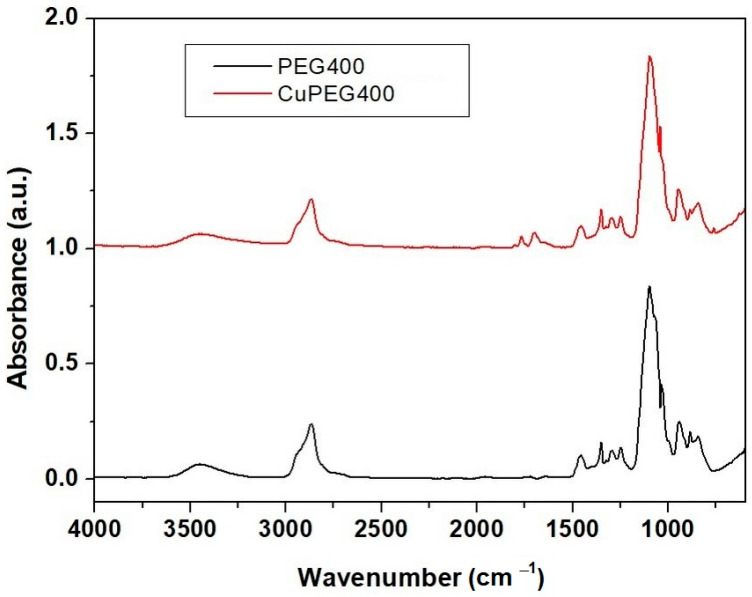
The FTIR-ATR spectra of Cu-PEG 400 and PEG 400.

For both samples from Figure 3, an absorption band around 3300 cm^−1^ was observed, which was due to the O–H stretching vibration. The band at 2867 cm^−1^ can be attributed to the stretching vibration of the aliphatic C–H group. The bands at 1768 and 1697 cm^−1^ are due to the stretching vibration of the C=O group and the one at 1091 cm^−1^ is attributed to the bonding of the C–C–O and C–C–H groups in the sample with Cu-PEG 400 clusters. From 1091 to 551 cm^−1^, the stretching vibrational modes of C–O and C–C groups are present [39]. The absorption bands at 1455 and 1347 cm^−1^ were due to the combined deformation vibrations of the O–C–H and C–O–H groups. At 1347 and 1249 cm^−1^, bands are due to the stretching vibration of the C–H and C–O groups. In fact, the bands due to the C–O stretching vibration were merged into the peaks at 1249 and 1062 cm^−1^, originating from the vibrations of the C–O, C–O–C, and C–O–H groups of the Cu clusters in PEG. Additionally, the bands at 1455 and 1349 cm^−1^ were due to C–H bending vibrations. The band at 1091 cm^−1^ is attributed to the bonding of the C–C–O and C–C–H groups. At small wavenumbers, absorption bands of unreacted ascorbic acid can be observed in the spectrum of Cu-PEG samples.

Therefore, FT-IR spectra showed the existence of van der Waals interactions between the PEG chain and copper derivatives in polymeric media [31,39].

**Figure 4:**
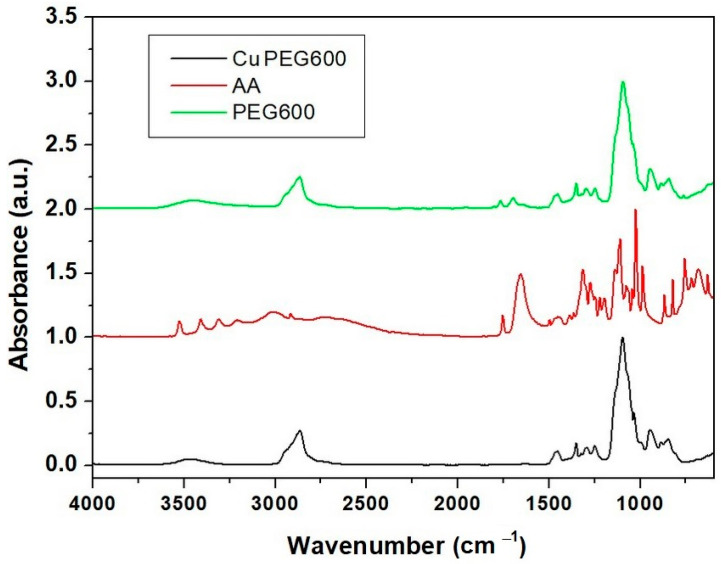
FT-IR spectra of copper clusters, ascorbic acid, and PEG 600.

From Figure 4, it can be observed that in the IR spectrum of CuPEG600, the absorption peaks specific to polyethylene glycol 600 predominate and the absorption peaks characteristic to ascorbic acid are almost completely missing. The lack of ascorbic acid characteristic peaks is due to its low concentration in the blends, on the one hand, and to its reaction with copper sulphate on the other hand.

In the spectrum of PEG 600, a wide absorption band can be observed at high wavelengths of about 3625–3322 cm^−1^ due to the presence of intra and intermolecular H bonding interactions O–O–H···O–H and O–H···O (oxygen of the ether group) [40,41], and stretching vibration of O–H group [42]. A C–H antisymmetrical stretching vibration of CH_2_ groups can be observed at 2865 cm^−1^ [39,40,42], while the band of absorption of C–H in plane-bending deformation of the same CH_2_ functional group can be noticed at 1455 cm^−1^ and 1350 cm^−1^ [39]. The absorption band at 1095 cm^−1^ can be attributed to stretching vibrations of O–H and C–O–H for the ether group (intermolecular H bonds). Absorption bands from 945 cm^−1^ and 845 cm-1 were generated due to CH_2_ stretch [42] or rocking [40], respectively.

In the spectrum of copper powder embedded in PEG600, the specific absorption bands of PEG can be noticed at the same wavenumbers, except that the C–H antisymmetrical stretch suffered a shift from 2865 cm^−1^ (in PEG600) to 2870 cm^−1^ in the sample of copper embedded in PEG 600 due to the presence of both copper and dehydroascorbic acid (C_6_O_6_H_6_). No absorption bands of ascorbic acid can be seen in the absorption spectrum of Cu PEG600 because ascorbic acid has been oxidized to dehydroascorbic acid, but new absorption peaks can be noticed at 1770 and 1700 cm^−1^. According to Jing Xiong et al. [11] and Shikha Jain et al. [43], the peaks correspond to the hydroxyl, oxidated ester carbonyl groups, and conjugated carbonyl groups, respectively.

The interaction between dehydroascorbic acid was seen through hydroxyl groups of ascorbic acid and dehydroacid, respectively, with copper particles stabilized [43].

#### 3.2.3. Ultraviolet-Visible Spectroscopy (UV-VIS)

**Figure 5:**
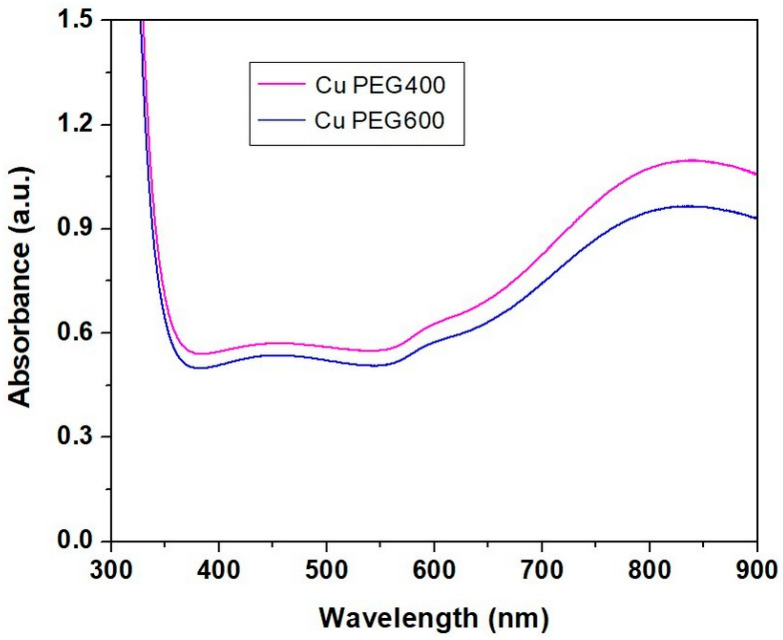
UV-VIS spectra of copper clusters obtained in PEG 400 and PEG 600, respectively.

For the UV-VIS spectra recording, the copper particles have been dispersed in glycerol. UV-VIS spectra of copper particles after 24 h (Figure 5) showed two absorption peaks, the first around 600 nm, and the second around 800 nm, together with a strong absorption peak in the UV domain. The absorption band from UV can be explained by the presence of organic compounds (PEG and glycerol) that absorb ultraviolet and can be attributed to the transitions between the nonbonding orbitals to the antibonding orbitals [11]. The peak around 837 nm appears due to the presence of nanometric copper powders. Other authors [11] observed absorption peaks at 802 nm for copper particles with an average size of 25.84 nm.

The increase of the absorbance of samples containing copper particles after the mixing of the reagents has been observed by Fathima et al. [4], with an increase in the reaction time. The wide surface plasmon resonance around 595 nm was developed due to the presence of copper nanoparticles with diameters less than 10 nm. Other authors obtained copper nanoparticles with diameters less than 4 nm, showing an absorption peak at 560 nm [44] or 6–10 nm with an absorption peak at 590 nm in the UV-VIS spectra [45].

### 3.3. Characterization of the PLA–Cu Samples

#### 3.3.1. Fourier Transform Infrared (FT-IR)

**Figure 6:**
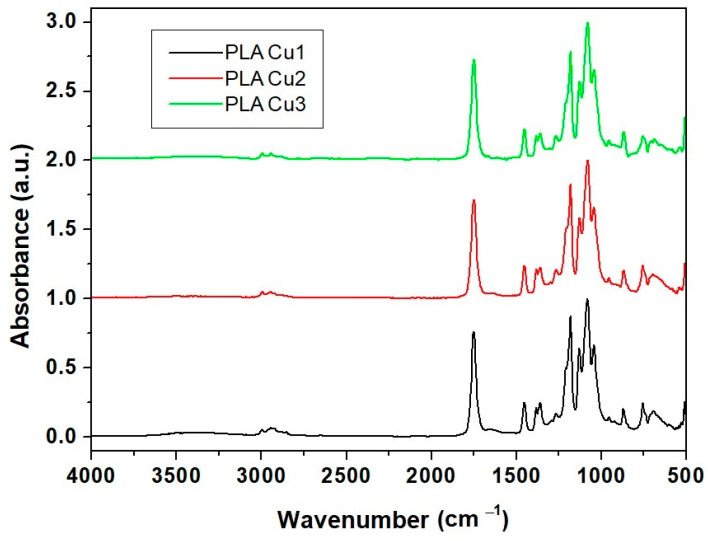
FT-IR spectra of PLA/Cu composites (samples PLACu1, PLACu2, PLACu3).

Figure 6 shows the FT-IR spectra of the composite samples containing copper obtained using PEG600. There are minor changes in the PLA spectrum due to the presence of the copper suspensions in PEG600 and Proviplast 2624, respectively. Small shifts in the absorption bands of polylactic acid following the preparation of the composite can be explained by the interactions between PLA and PEG and PLA and Proviplast 264, respectively. Table 2 presents the main absorption bands of PLA and PLA-based composites.

#### 3.3.2. Tensile Strength Testing

**Figure 7:**
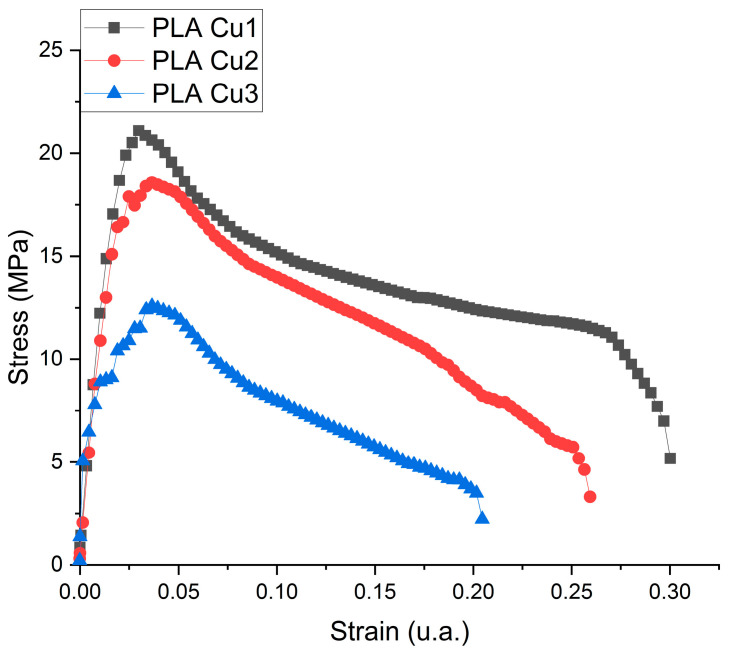
Tensile deformation curve of the PLA-Cu composite sample.

The tensile deformation curves for the PLA-Cu composites are presented in Figure 7 and the results obtained for tensile strength tests are in Table 3.

It is known from specialized literature that the higher the amount of PLA %, the higher the tensile strength and Young’s modulus [46]. Comparing the PLA Cu1, PLA Cu2, and PLA Cu3 samples, in which the difference is the ratio between polylactic acid and PEG 600-Cu mixture, it can be seen that the tensile strength of the samples is directly proportional to the ratio between the two components. One can observe the similar behavior of the samples to the force to which it was subjected, with the elongation of each sample decreasing once met with resistance.

PLA has a rigid but brittle polymer chain. Its strength (59.1 MPa) decreases with the introduction of dispersed additives in its polymer matrix. Therefore, there are numerous reports in the literature about the dispersion of these additives, either in certain plasticizers [47] or in elastic fillings [48]. However, it is essential not to exceed certain additive concentrations. Mastalygina and co. [49] are of the opinion that the addition of 5–10 wt % of copper (II) sulfate to the PLA leads to a significant decrease in tensile strength due to the tendency of particles to agglomerate in the PLA melt. By adding PEG, as a plasticizer, there is an increase in elongation relative to breaking, thus increasing the modulus of elasticity. Therefore, Cu has a better dispersion affinity in PEG, which has a low molecular weight, reducing the formation of agglomerates in the polymer melt.

#### 3.3.3. Flexural Strength Testing

**Figure 8:**
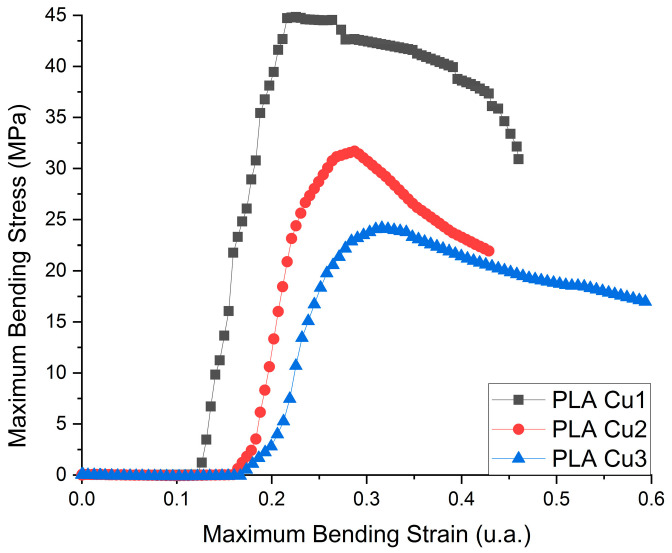
Flexural deformation curve of the PLA-Cu composite sample.

The flexural deformation curves of the PLA-Cu composite samples are presented in Figure 8 and the obtained results for three points Flexural Tests are in Table 4.

From flexural test investigation, we can deduce that the flexibility of the samples decreases with a decrease in the amount of PLA. The Young’s modulus of the samples is directly proportional to the maximum force supported and is inversely proportional to the elongation. As in the case of tensile tests, the sample with the highest amount of PLA has the highest resistance and supported force, but the lowest elongation, being stiffer than the samples with higher PEG 600-Cu contents.

The Cu clusters show catalytic activity in the process of converting the ester groups from the recipes into hydroxyl groups, reactions that decrease the average molecular mass of the recipe. The increase in the content of Cu clusters favors the increase in the content of more volatile compounds in the sample during the preparation process of PLA Cu samples and, implicitly, the mechanical properties (tensile, flexural strength) of the composites were decreased.

#### 3.3.4. Differential Scanning Calorimetry (DSC)

**Figure 9:**
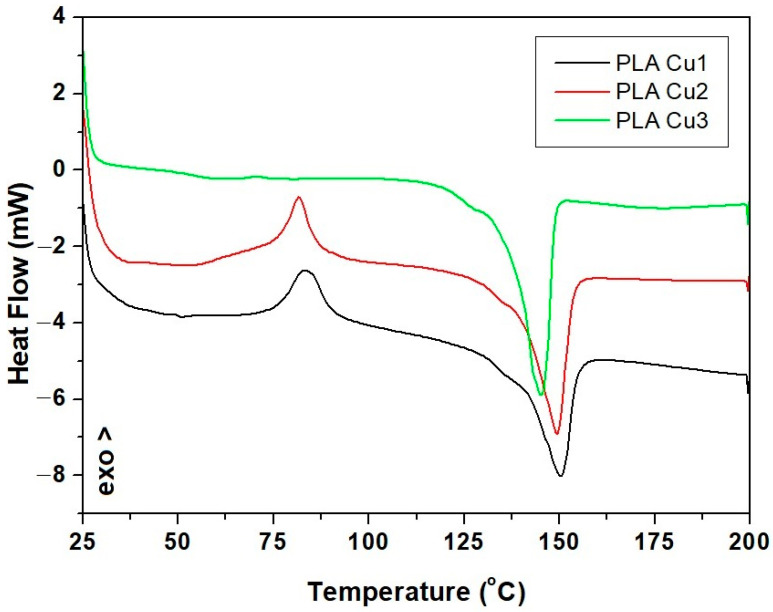
DSC curves of PLA-Cu samples.

With the increase in temperature, phase transitions occur, which involve a change in the way the particles are arranged and a change in the thermodynamic properties of the material. Table 5 and Figure 9 show the temperature ranges in which transformations take place in samples and the identification of exothermic or endothermic processes.

Through the analysis of the three DSC curves (Figure 9, Table 5), corresponding to the analyzed samples, it was observed that the glass transition, crystallization, and melting temperature decrease with the increase in the percentage of Cu-PEG600, respectively, and with the decrease in the content of PLA in the sample’s composition due to the decrease of molecular weight with the increase of Cu-PEG600 concentration. Copper clusters act as catalysts in the reaction of PLA decomposition, affecting the thermal properties of PLA-Cu composites. On the other hand, the dispersed Cu clusters can act as nucleating agents, leading to a heterogeneous PLA crystallization [50].

For the PLA-Cu1 sample, in the temperature range 25–100 °C, an exothermic process was identified, with transformation temperature values of 83.41 °C. In the temperature range 100–200 °C, an endothermic process (melting) was identified, with the onset of transformation temperatures of 136 and 149.99 °C, respectively.

The increase of the concentration of CuPEG600 led to the decrease of the glass transition temperature to 81.59 for sample PLACu2 and 70.72 for the PLACu3 sample. An important decrease in melting and crystallization temperature took place due to an increase in CuPEG600 content (Table 5).

#### 3.3.5. Thermogravimetric Analysis (TGA)

**Figure 10:**
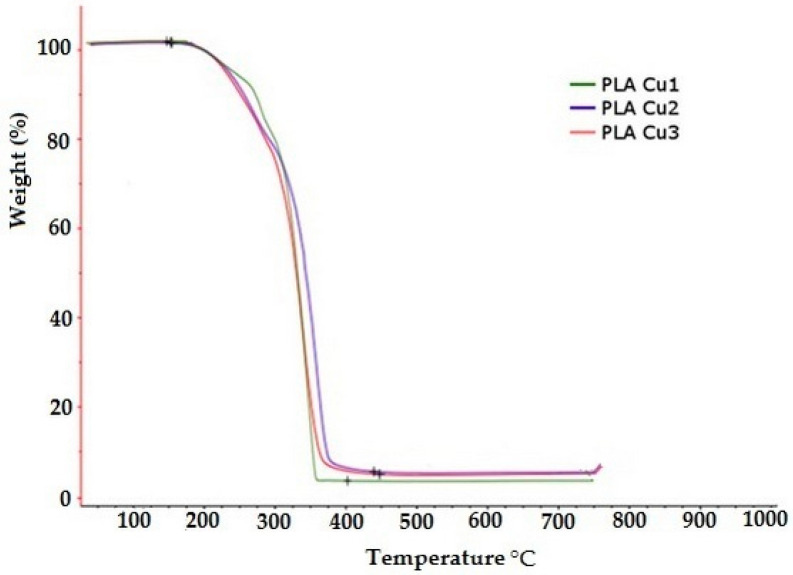
Thermogravimetric analysis (TGA) for the PLA-Cu samples.

The thermal decomposition of PLA Cu samples is presented in Figure 10. Two important areas are observed: (1) an area with a mass loss of up to 15% by weight, the area located at temperatures of up to approximately 270 °C which corresponds mainly to mass losses through the vaporization of water and plasticizer, and (2) the area with mass losses greater than 15% by weight, and the area located at temperatures above 270 °C which corresponds to the degradation of the mixture components (see Table 6). It is observed that the temperature at which mass losses are 15% by weight decreases with increasing content of Cu clusters in the PLA recipe. This behavior is probably due to the catalytic action of the Cu clusters in the conversion process of the ester groups from the sample to hydroxyl groups, reactions that decrease the average molecular mass and favor the increase of losses through vaporization. Thus, the increase in the content of Cu clusters favors the increase in the content of more volatile compounds in the sample during the preparation process of PLA Cu recipes and, implicitly, the decrease in the temperature at which mass losses are 15% by weight.

The crystalline phase of the PLA composites depends on the processing conditions and on the type of plasticizers, but also on the nucleating agents [32].

#### 3.3.6. Absorption of the Saline Solution (%)

High water absorption could accelerate the degradation rate of PLA. However, PLA has a hydrophobic character, due to the methyl groups (–CH_3_), which are non-polar covalent groups that are water-resistant due to the steric shielding effect [51,52]. An increase in the hydrophilic properties of PLA was attempted by adding starch which led to a disruption of the crystalline structure of PLA, accelerating the degradation of PLA blends. It was found that the hydrolysis in the crystalline region is much slower than that in the amorphous region, where water can penetrate more easily, not having an organized structure like the crystalline one [52]. Therefore, the degree of crystallinity of PLA can be modified by adding certain additives, obtaining different degradation rates.

**Figure 11:**
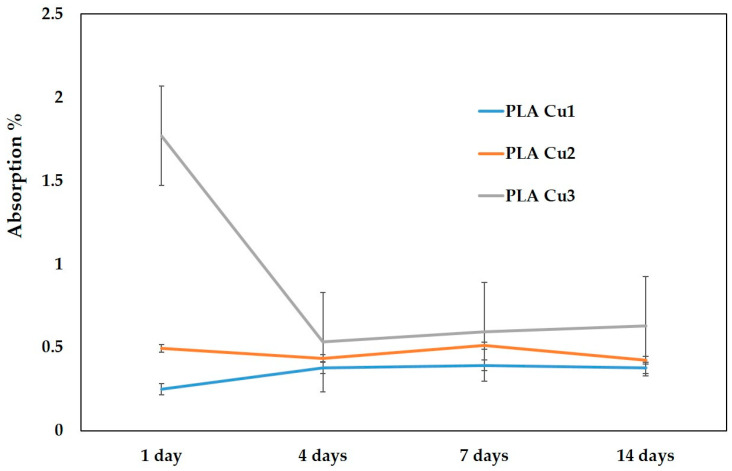
Absorption of the saline solution (%).

From Table 7 and Figure 11, it can be seen that for the PLA-Cu3 sample (with 3% Cu PEG600) the highest absorption values were recorded throughout the entire storage period of the samples in saline solution. The lowest absorption values were recorded in the case of the sample containing 1% Cu- PEG600 (PLA-Cu1). After 4 days, and until the end of the investigation period, the absorption values of the three samples are quite close, slightly higher, and after 7 and 14 days, respectively, for the PLA-Cu3 sample.

Comparing the evolution of the three samples, there are no statistically significant differences between the absorption values in the 14 days of measurement (*p* = 0.12751).

#### 3.3.7. Contact Angle

**Figure 12:**
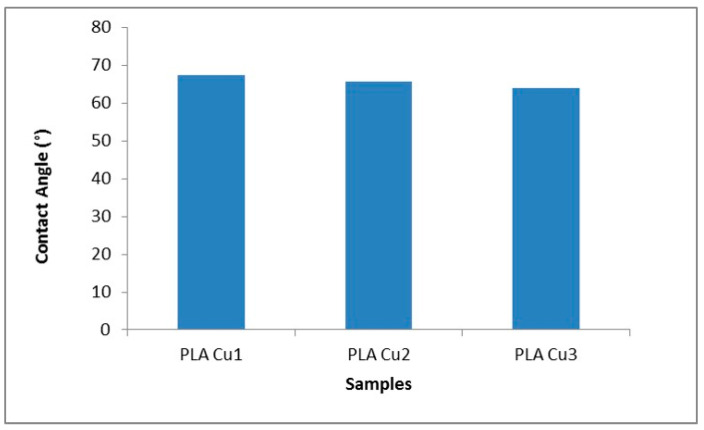
Contact angle values for PLA 600-Cu composites.

The contact angle values are shown in Figure 12; it was observed that it has relatively high values, probably due to the high hydrophobicity of the plasticizer as well as of the PLA. At the same time, the contact angle decreases with the increase in the concentration of the PEG 600-Cu mixture in the recipes. These results suggest that the hydrophilicity of PEG 600 and the Cu clusters improved the polarity of the recipes by favoring the formation of hydrogen bonds between the surface of the recipes and water molecules. The same behavior was observed by Pulgarin et al., who showed that the polar groups of the Tween 20 molecule favored the increase of the hydrophilicity of the material [53].

#### 3.3.8. Scanning Electron Microscopy of PLA Cu Sample

**Figure 13:**
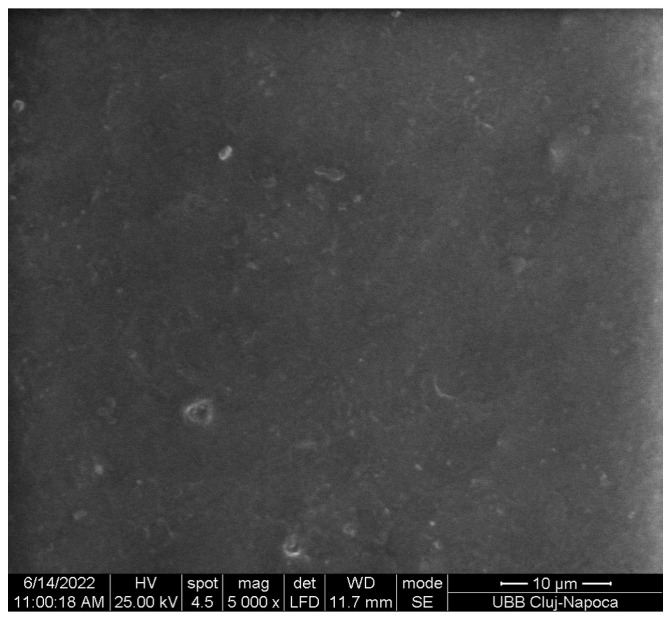
SEM image ×5000 magnification on the PLA-Cu1 sample surface.

Increasing the amount of copper clusters increases the elongation and decreases the Young’s modulus and the maximum load, being normal for the composites with particle size in microns, which supposes that agglomerates of Cu clusters formed. The SEM image of Figure 13 present agglomerates, and the surface is not entirely smooth. The small particles that stand out can also come from the sample that was processed and cut.

#### 3.3.9. Antimicrobial Activity

**Figure 14:**
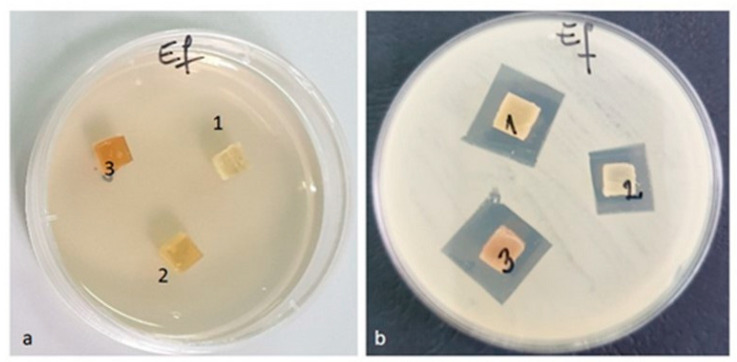
*Enterococcus faecalis* ATCC 29212 (**a**) zero (initial) moment, (**b**) inhibition after 48 h of incubation.

**Figure 15:**
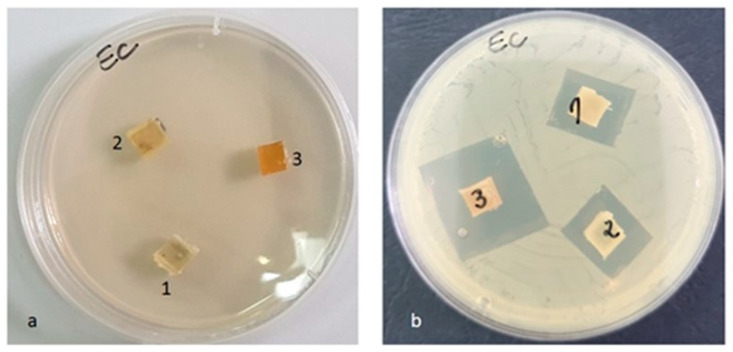
*Escherichia coli* ATCC 25922 (**a**) zero (initial) moment, (**b**) inhibition after 48 h of incubation.

**Figure 16:**
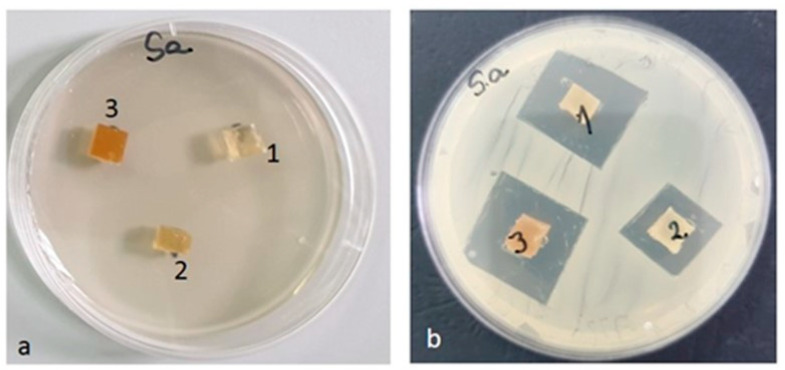
*Staphylococcus aureus* ATCC 25923 (**a**) zero (initial) moment, (**b**) inhibition after 48 h of incubation.

**Figure 17:**
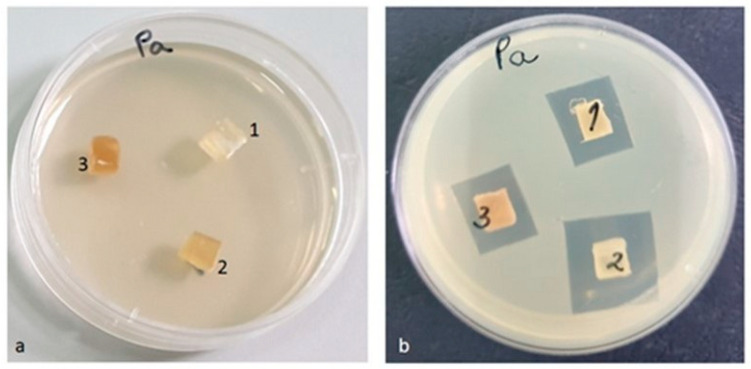
*Pseudomonas aeruginosa* ATCC 27853 (**a**) zero (initial) moment, (**b**) inhibition after 48 h of incubation.

After the end of the incubation period, at 37 °C, the zones of inhibition (mm) were determined for the tested microbial strains. It was observed that in all the tested samples, the size of the zone of bacterial inhibition varied against all the bacterial strains studied according to the type of material and according to the tested microbial strain (Table 8). The numbering of the samples (with 1, 2, 3) in the Figure 14, Figure 15, Figure 16 and Figure 17 corresponds to the samples code and can be seen in the Table 8.

Against the bacterial *Enterococcus faecalis* ATCC 29212 strain, an inhibition was observed in all three tested samples (Figure 14). The lowest inhibition of this strain was recorded by the PLACu1 sample (14 mm) (Table 8).

Against the *Escherichia coli* ATCC 25922 strain, an inhibition zone was observed for all samples, but the highest was in the case of the PLACu3 sample (22 mm) (Figure 15), containing the higher concentration of copper.

Against the *Staphylococcus aureus* ATCC 25923 strain, the inhibition zone was observed in all three composite samples. The highest inhibition was recorded for the PLACu3 sample (22 mm), followed by the PLACu2 sample (20 mm). The PLACu1 sample showed only an inhibition zone of 14 mm (Figure 16). In the case of *Staphylococcus aureus*, the size of the inhibition zone depends strongly on the content of copper.

In contrast to the other tested strains, against the *Pseudomonas aeruginosa* ATCC 27853 bacterial strain, the highest inhibition by the PLACu1 sample (20 mm) was recorded. In the other two samples, the diameter of the inhibition zone was quite similar (Figure 17).

**Figure 18:**
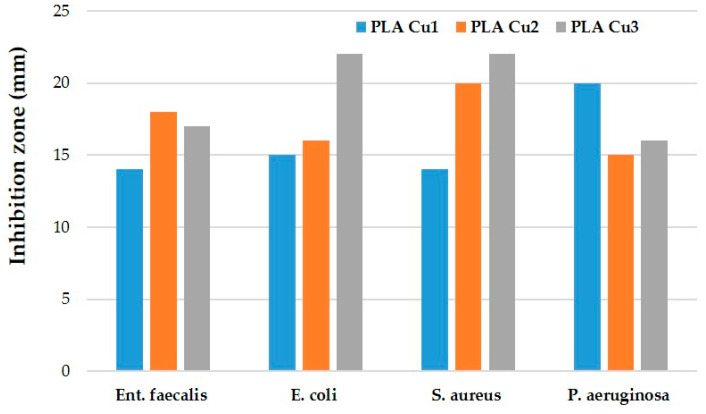
The antibacterial effect of the tested samples.

To summarize, the bacterial inhibition was recorded by all samples and varied depending on the sample and the tested bacterial strain. However, the lower values were presented by the PLACu1 (sample 2) with the smallest amount of copper, except the one, against *Pseudomonas aeruginosa* (pyocyanin bacillus, Gram-negative) (Figure 18).

Antimicrobial and antifungal properties of copper particles against *E. coli*, *S. aureus,* and *Alternariaspecies*, have been previously demonstrated [54], and depend on copper concentration.

Other authors found that composites containing polylactic acid and copper were active against *P. fluorescens* and *P. putida* [55].

According to [56], copper interacts with groups from the outer membrane or cell wall of bacteria, determining the denaturation of proteins. Copper also interacts with N-acetylglucosamine and N-acetylmuramic acid in the peptidoglycan layer. Following those interactions, the membrane of bacteria disintegrates. After entering the bacterial cells, copper binds DNA, determining the destruction of helical structures due to crosslinking interactions, interrupting the replication cycle of the bacteria. Copper is also involved in the generation of reactive oxygen species, determining protein and lipid oxidation.

## 4. Conclusions

Copper clusters have been obtained by chemical reduction of Cu (II) ions from CuSO_4_ with ascorbic acid using PEG as a dispersion environment, applying reactive milling. Following the reaction between CuSO_4_ and ascorbic acid, clusters of copper particles have been obtained. The use of PEG 600, as a plasticizer, was chosen for the dispersion of Cu particles, for achieving a high adhesion between the hydrophilic additive particles and the hydrophobic polymer, in order to decrease the additive’s agglomeration tendency and to improve the physical and mechanical properties of the material.

Copper clusters have been investigated by UV-VIS spectroscopy, showing the absorption bands of copper. FT-IR spectroscopy revealed the presence of absorption bands of PEG and unreacted ascorbic acid in the spectrum of copper due to the presence of PEG as a dispersion environment. FT-IR spectra of the PLA-based composites containing copper showed small shifts in the bands of PLA due to the interactions between PLA, PEG, and the plasticizer Poviplast.

By DSC analysis, it was observed that the transformation temperatures of samples decrease, with an increase in the percentage of added additives. The increase in the content of Cu clusters leads to the decrease of average molecular weight of PLA, leading to a decrease in transition temperatures. The highest absorption of saline solution values were recorded for sample with 3% Cu throughout the entire investigation period. The contact angle decreases with the increase in the concentration of the Cu-PEG 600-mixture in the samples, improving the polarity of the recipes and forming hydrogen bonds between the surface of the composite samples and the water molecules.

Mechanical testing was conducted to the conclusion that the tensile strength, young modulus, breaking elongation, and bending stiffness of the samples decreased with the increase of Copper/PEG concentration.

The obtained composite showed antibacterial properties for all tested strains (*Enterococcus faecalis, Escherichia coli, Staphylococcus aureus*, and *Pseudomonas aeruginosa*).

## Figures and Tables

**Table 1:** The composition of the samples and the obtaining conditions.

Sample No.	Polylactic Acid [wt%]	Proviplast 2624 [wt%]	PEG 600 + CuSO_4_ [wt%]
PLA Cu1	84	15	1
PLA Cu2	83	15	2
PLA Cu3	82	15	3

**Table 2:** The attribution of the main absorption bands from the spectra of PLA and PLA-based composites.

PLACu1	PLACu2	PLACu3	Attribution
870	868	868	C–C stretching
1043	1043	1043	C–CH_3_ stretching
1180 1130 1082	1180 1128 1085	1182 1128 1079	C–O–C asymmetrical stretching
1265	1265	1267	C=O bending
1383 1359	1383 1359	1383 1359	CH δ (asymmetrical and symmetrical)
1359			
1454	1454	1452	CH_3_ δ asymmetrical
1749	1749	1749	C=O stretching

**Table 3:** The results obtained for tensile strength tests.

Samples Code	Tensile Strength (MPa)	The Highest Reached Force [N]	Breaking Strength [N]	Young Modulus [MPa]	Breaking Elongation [mm]	Breaking Stress [MPa]
PLA Cu1	20.6358 ± 2.03341	224.2592 ± 15.92412	44.8518 ± 0.54774	1322.8194 ± 663.43933	10.3244 ± 1.07951	4.1271 ± 0.49338
PLA Cu2	16.8036 ± 0.49944	116.1344 ± 9.11474	25.2663 ± 0.46761	904.5661 ± 42.74934	9.8474 ± 1.35432	6.8084 ± 0.48921
PLA Cu3	12.9715 ± 1.67583	109.5749 ± 5.68169	22.8479 ± 1.90622	874.1094 ± 75.00176	5.79814 ± 0.47594	10.4781 ± 0.5244
*p*	0.00199	2.61422 × 10^−5^	6.0359 × 10^−7^	0.97324	0.00749	2.03095 × 10^−5^

**Table 4:** The obtained results for three points Flexural Tests.

Samples Code	Maximum Load [N]	Young Modulus [MPa]	Bending Stiffness [Nm^2^]	Maximum Bending Stress at Maximum Load [MPa]	Elongation [mm]
PLA Cu1	104.7791 ± 2.82514	874.9952 ± 56.19848	0.032 ± 0.003	45.7740 ± 4.30885	5.5509 ± 0.50416
PLA Cu2	68.6247 ± 1.76737	722.0915 ± 30.34548	0.022 ± 0.00569	29.5459 ± 3.56033	5.828 ± 0.54864
PLA Cu3	68.4348 ± 1.00393	420.3860 ± 15.23471	0.012 ± 0.002	24.8904 ± 2.08914	7.6838 ± 0.23053
*p*	9.8591 × 10^−7^	2.13035 × 10^−5^	0.00221	7.4621 × 10^−4^	0.00156

**Table 5:** The phase transformation temperatures and the identification of exothermic or endothermic processes.

Sample	Amount [mg]	DSC		Process
		Temperature Range [°C]	The Phase Transformation Temperatures [°C]	
PLA Cu1	10.466	25–100	83.41	exothermic
		100–200	Onset~136 149.99	endotherm
PLA Cu2	10.430	25–110	81.59	exothermic
		110–200	Onset 138.28 149.14	endotherm
PLA Cu3	11.259	25–90	70.72	exothermic
		90–200	Onset 125.88 144.93	endotherm

**Table 6:** The temperature at which the sample loses 15% by weight.

Sample	Temperature for Mass Loss of 15% by Weight, °C
PLA-Cu1	287
PLA-Cu2	273
PLA-Cu3	271

**Table 7:** Absorption values and ± standard deviation according to the immersion time of the samples.

Sample	Immersion Time				
	1 Day	4 Days	7 Days	14 Days	*p*
PLA Cu1	0.24824 ± 0.02446	0.37502 ± 0.04044	0.39234 ± 0.01594	0.37594 ± 0.00909	0.01388
PLA Cu2	1.76949 ± 2.0064	0.53089 ± 0.00182	0.59365 ± 0.01568	0.62693 ± 0.00283	0.59862
PLA Cu3	0.49399 ± 0.17411	0.43393 ± 0.04571	0.5106 ± 0.05717	0.42293 ± 0.0163	0.74927

**Table 8:** The diameter of the bacterial inhibition zones (mm) of the tested samples.

Sample Numbering/Sample Code	1	2	3
	PLA Cu2	PLA Cu1	PLA Cu3
Bacterial strain			
*Enterococcus faecalis* ATCC 29212	18	14	17
*Escherichia coli* ATCC 25922	16	15	22
*Staphylococcus aureus* ATCC 25923	20	14	22
*Pseudomonas aeruginosa* ATCC 27853	15	20	16

## Data Availability

Not applicable.
